# Undergraduate medical students’ perceptions, attitudes, and competencies in evidence-based medicine (EBM), and their understanding of EBM reality in Syria

**DOI:** 10.1186/1756-0500-5-431

**Published:** 2012-08-12

**Authors:** Fares Alahdab, Belal Firwana, Rim Hasan, Mohamad Bassam Sonbol, Munes Fares, Iyad Alnahhas, Ammar Sabouni, Mazen Ferwana

**Affiliations:** 1 University of Damascus, Faculty of Medicine, Damascus, Syria; 2 Department of Internal Medicine, University of Missouri, Columbia, MO, USA; 3 National and Gulf Center for Evidence Based Health Practice, King Saud bin Abdulaziz University for Health Sciences, Riyadh, Saudi Arabia

## Abstract

**Background:**

Teaching evidence-based medicine (EBM) should be evaluated and guided by evidence of its own effectiveness. However, no data are available on adoption of EBM by Syrian undergraduate, postgraduate, or practicing physicians. In fact, the teaching of EBM in Syria is not yet a part of undergraduate medical curricula. The authors evaluated education of evidence-based medicine through a two-day intensive training course.

**Methods:**

The authors evaluated education of evidence-based medicine through a two-day intensive training course that took place in 2011. The course included didactic lectures as well as interactive hands-on workshops on all topics of EBM. A comprehensive questionnaire, that included the Berlin questionnaire, was used to inspect medical students’ awareness of, attitudes toward, and competencies’ in EBM.

**Results:**

According to students, problems facing proper EBM practice in Syria were the absence of the following: an EBM teaching module in medical school curriculum (94%), role models among professors and instructors (92%), a librarian (70%), institutional subscription to medical journals (94%), and sufficient IT hardware (58%). After the course, there was a statistically significant increase in medical students' perceived ability to go through steps of EBM, namely: formulating PICO questions (56.9%), searching for evidence (39.8%), appraising the evidence (27.3%), understanding statistics (48%), and applying evidence at point of care (34.1%). However, mean increase in Berlin scores after the course was 2.68, a non-statistically significant increase of 17.86%.

**Conclusion:**

The road to a better EBM reality in Syria starts with teaching EBM in medical school and developing the proper environment to facilitate transforming current medical education and practice to an evidence-based standard in Syria.

## Background

Innovation in information technology alongside massive increase in biomedical research has given rise to a relentlessly changing biomedical literature varying in quality and clinical relevance. This has led to the emergence of evidence-based medicine (EBM) as the new paradigm for medical practice [1].

EBM is defined as the “conscientious, explicit, and judicious use of current best evidence.” [2] It involves integrating individual clinical expertise with the best available external clinical evidence and use of individual patients’ values and preferences in making clinical decisions about their care [3]. The term EBM entered the lexicon in 1992. Since then, it has become the latest focus in the search for improved health care [4].

EBM has thus become an impetus for incorporating critical appraisal of research evidence alongside routine clinical practice. Increasingly, acquisition of knowledge and skills for EBM is becoming a core competence to be acquired by all doctors. As a result of studies demonstrating substantial gaps between research evidence and the care provided in usual clinical practice [5], there is an increasing emphasis on the teaching of EBM skills in undergraduate, postgraduate, and continuing medical education programs [6-9].

Teaching of EBM should be evaluated and guided by evidence of its own effectiveness. Therefore, many studies have been conducted to assess the awareness of, attitudes toward, and competencies in EBM in medical students as well as in the general practice [10-16]. However, no data are available about the adoption of EBM by Syrian undergraduate, postgraduate, or practicing physicians. In fact, teaching EBM in Syria is not yet a component of the undergraduate medical curricula.

We aimed to evaluate students’ level of education of EBM though a two-day intensive training course. We also aimed to explore the feasibility of introducing an EBM course to improve students’ competencies in EBM into the medical school curriculum.

## Methods

### Study design and participants

A within-subjects study design with pre- and post-course tests and questionnaires was used. Undergraduate medical students at Damascus University, Faculty of Medicine, in Damascus, Syria, participated voluntarily in the study. The Faculty of Medicine of Damascus University is the first, largest, and by far the most prominent faculty of medicine in Syria. It attracts students from all provinces of Syria as well as from neighboring countries such as Lebanon, Iraq, and Jordan. The Medical Diploma (MD) program at Damascus University Faculty of Medicine is a six-year program consisting of three basic science (preclinical) years and three clinical years. A verbal consent was taken from all participants. The Review Committee of Damascus University Faculty of Medicine has revised and approved the protocol of the study.

### Educational intervention

An EBM course was given by a core group of experienced medical students and recently graduated physicians certified as ‘EBM Instructors’ by the National and Gulf Center for Evidence-Based Health Practice (NGCEBHP). The EBM course comprised a series of interactive large and small group seminars and practical sessions. The curriculum outline of the course included the following: definition of EBM, formulating focused, answerable clinical questions, developing and carrying out effective search strategies, accessing EBM resources, critically appraising different types of research evidence including therapeutic randomized controlled trials (RCTs), diagnostic RCTs, prognostic studies, and systematic reviews, and understanding clinically-relevant basics in biostatistics. A lecture on developing and utilizing guidelines of therapy and diagnosis was also included.

The different components constituting our course curriculum had clear and specific educational objectives. In “Definition of EBM” sessions, the attendees were introduced to the concept of EBM and how to use it in clinical practice. They were taught to recognize situations that impose clinical uncertainty, and whether a background or foreground question was to be answered to resolve this uncertainty. Afterwards, they were exposed to the PICO trend (P: population or patient; I: intervention; C: comparison; and O: outcome) in deriving highly specific clinical questions from clinical problems [17-19]. They were instructed to utilize the PICO question to develop successful strategies in searching for evidence in medical databases. Taking into consideration the limited resources available online at Damascus University and its affiliated hospitals, the students were taught to use only the PubMed and Cochrane databases.

The largest component of the curriculum was dedicated to teach the students how to critically evaluate and appraise clinical evidence. They learned methods for the critical appraisal of therapeutic and diagnostic RCTs and when to deem a study relevant, valid, useful, and applicable. They were also taught how to analytically evaluate a systematic review or meta-analysis to reach a final judgment. The critical appraisal sessions stressed the most clinically relevant concepts in biostatistics, especially: absolute risk reduction, relative risk reduction, number needed to treat, interpretation of confidence intervals, sensitivity and specificity, positive and negative predictive values, and likelihood ratios. In addition to the above-mentioned biostatistical concepts emphasized during the appraisal sessions, an independent session was included dedicated to the review of the basics of biostatistics. Unlike many other EBM courses, a session was added to introduce students to the methods used to develop clinical guidelines, in order to better equip the students to link evidence with decision-making at the point of care.

### Outcome assessment

Participation in the EBM course was voluntary. The students completed an identical set of pre- and post-test questionnaires, which included an unmodified version of the Berlin questionnaire [20,21], before the beginning of the first session of the course and after the students finished the last session of the course. The test is composed of 15 multiple-choice questions, each receiving one mark, for a complete score of the test 15 marks. Remaining questions in the questionnaire were aimed at inspecting the students’ self-reported perceptions of their competencies in EBM, their attitudes toward EBM, and their receptiveness to the idea of adding regular education of EBM into the medical school curriculum.

### Data analysis

All data was entered into a Microsoft Excel spreadsheet and exported for analyses using the statistical software SPSS v17.0 (Chicago, IL, USA). The chi-square test and Fisher’s exact test were used for comparing categorical variables, and students’ t-test was used for comparing continuous variables. As multiple comparisons were made, probability values of <0.05 were taken to denote statistical significance. Standard approaches to summarize questionnaire data were used, including frequencies and descriptive summaries for categorical data, and means, ranges, standard deviations and 95% confidence intervals for numerical data.

## Results

### Participants

All 50 undergraduate students who attended the course completed the questionnaires (response rate of 100%). Basic characteristics of participants are describes in Table 1.

**Table 1:** Participants’ Characteristics

***Variables***		***N***	***%***
***Gender***	Male	29	58
	Female	21	42
***Educational level***	Basic science (pre-clinical) years	12	24
	Clinical years	38	76
***Previous EBM exposure***	Read about or attended a lecture	28	56
	Had a formal EBM training	0	0
***Previous research exposure***	Participated in ≥ 1 research activity	31	62
	No previous research exposure	19	38
***Frequency of online search for evidence***	≥ once per week	16	32
	< once per week, or not at all	34	68
***First source sought for evidence (before the course)***	Google	23	46
	UpToDate^TM^	13	26
	PubMed	7	14
	The Cochrane library	1	2
	Other	3	6

The assessment of the participants’ previous exposure to any training in evidence-based medicine showed that 28 students (56%) had read about or attended a lecture on EBM.

### Objective Evaluation of EBM Skills before and after the Course

The mean score of the students’ pre- and post-course Berlin tests were 5.6/15 and 8.28/15, respectively. Therefore, the objectively measured increase in EBM knowledge and skills was 2.68 (95% CI: 1.68 - 3.68; p-value > 0.05) which is a non-statistically significant increase of 17.86%. Interestingly, female attendees had better post-test scores than males (9.5 and 7.9 respectively; p-value = 0.02), and a higher gain in EBM knowledge (26% and 12% respectively; p-value = 0.031).

### Perceived knowledge in EBM before and after the course

**Figure 1:**
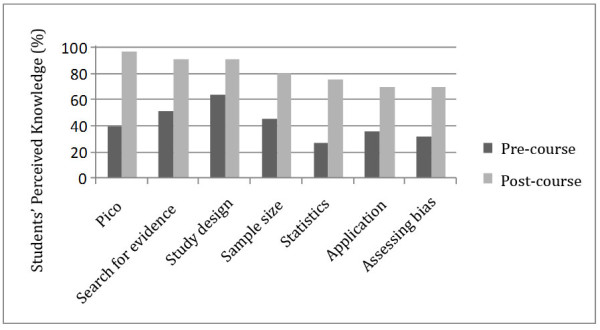
Medical students’ self-reported confidence in different components of EBM before and after the course.

As part of our assessment of the efficacy of the learning module we used in our EBM course, we aimed to measure the increase in self-reported understanding and knowledge of the different concepts of EBM. Thus, our questionnaires included a set of questions about the self-reported ability of medical students to go through the steps of EBM in both pre- and post-course questionnaires. Figure 1 shows medical students’ self-reported confidence in different components of EBM before and after the course, and Table 2 summarizes this perceived knowledge before and after the course.

**Table 2:** Perceived knowledge in EBM before and after the course

***EBM Knowledge Components***	***Easy/Relatively Easy***				***Difficult/Relatively Difficult***			
	**Pre**		**Post**		**Pre**		**Post**	
***PICO***	21	(41%)	49	(98%)	30	(60%)	2	(3%)
***Search***	26	(52%)	46	(92%)	24	(48%)	4	(8%)
***Study design***	33	(65%)	46	(92%)	18	(35%)	4	(8%)
***Bias***	16	(32%)	36	(71%)	34	(68%)	15	(30%)
***Sample size***	23	(46%)	41	(81%)	27	(54%)	10	(19%)
***Applicability***	14	(27%)	32	(63%)	37	(73%)	19	(38%)
***Statistics***	14	(28%)	38	(75%)	38	(76%)	13	(25%)
***Point of care***	18	(36%)	36	(71%)	32	(64%)	15	(30%)

Students’ self-perceived ability of formulating PICO questions showed that 40.4% thought they could do so easily or relatively easily before taking the course, compared to 97.3% after the course, an impressive statistically significant increase of 56.9% (P < 0.001). When it came to the students’ skill of searching online for relevant evidence, 52.1% reported this was easy or relatively easy before the course, compared to 91.9% after the course, an increase of 39.8% (p-value < 0.001). Questions about skills in evaluating studies for strengths and weaknesses before and after the course showed an impressive increase in self-confidence. For instance, students’ self-assessment of their ability to assess the suitability of study designs easily or relatively easily had increased by 27.3% (p-value < 0.01). When asked about sample size calculations prior to the course, students thought themselves as being able to easily or relatively easily decide if a particular sample size was suitable for a study, which increased by 35.3% upon finishing the course (p-value < 0.01). In regards to statistics, a usually difficult area for students, only 27.7% before the course reported they could handle statistics of research easily or relatively easily, but after taking our course the percentage increased to 75.7%, a 48% increase (p-value < 0.001). When the students were asked about their abilities in applying the evidence at the clinical point of care, 36.2% before the course, compared to 70.3% after the course, thought this was easy or relatively easy, an increase of 34.1% (p-value < 0.01). Regarding the students’ confidence in assessing biases in research studies, 31.9% of students before the course, compared to 70.3% after the course thought it was easy or relatively easy, an increased in confidence of 38.4% (p-value < 0.001) is reported. Overall, when the students were asked about their confidence in evaluation of evidence, 34% of students thought it was easy or relatively easy in the pre-test, compared to 83.8% after the course, a statistically significant increase of 49.8% (p-value < 0.001).

### Comparison between objectively- and subjectively-measured gain in EBM knowledge

The mean increase in self-reported knowledge and confidence in EBM concepts mentioned above was 28%, compared to 17.86% increase in Berlin test scores. This suggests that the students tended to overestimate their skills in EBM after completing the course.

### Attitudes toward EBM before and after the course

Students’ answers to questions probing their attitudes toward EBM before and after the course showed that the course corrected many of their previous false impressions they had about EBM. Details of the students’ attitudes toward EBM are summarized in Table 3. When asked if EBM had a weak effect on the practice of medicine, 20% of the students before taking the course agreed with this statement, while after the course this percentage dropped to 8%. Some confusion regarding the relationship between EBM and the process of clinical decision-making was found in the medical students’ answers before taking the course, with 53% of them saying that clinical experience was more important than evidence. However, after the course only 19% still agreed with that statement. As for whether patient desire should override the evidence, confusion among students’ before the course did not change significantly after the course, with 42% and 36%, respectively, said that it should. However, an interesting between-gender difference was reported in deciding if patient desires are more important than medical evidence, with 46.2% of males, compared to 38.9% of females, agreeing with this statement, but this difference was not statistically significant (p-value > 0.05).

**Table 3:** Attitudes toward EBM before and after the course (with N &%)

***EBM Attitude Component***	***AGREEMENT***				***DIS-AGREEMENT***			
	**Pre**		**Post**		**Pre**		**Post**	
***Expertise***	27	(53%)	10	(19%)	23	(46%)	41	(82%)
***Need training***	49	(98%)	50	(100%)	0	(0%)	0	(0%)
***Curriculum***	50	(100%)	50	(100%)	0	(0%)	0	(0%)
***I can assess***	17	(34%)	42	(84%)	34	(67%)	8	(16%)
***Is Systematic reviews important in decision-making***	47	(94%)	50	(100%)	3	(6%)	0	(0%)
***EBM had a weak effect on medical practice***	10	(20%)	4	(8%)	40	(80%)	46	(92%)
***Good training***	12	(24%)	46	(92%)	38	(76%)	4	(8%)
***Patients desire override evidence***	21	(42%)	18	(36%)	29	(58%)	32	64%)
***EBM trend***	8	(15%)	0	(0%)	43	(85%)	50	(100%)
***Do research***	48	(96%)	50	(100%)	2	(4%)	0	(0%)
***Do not have the ability***	26	(52%)	19	(38%)	24	(48%)	31	(62%)

When asked if EBM was merely a passing fashion and that it would disappear soon, 16% of the students agreed with this statement before the course, but none of them agreed with it after taking the course.

A slight change in medical students’ attitudes toward EBM was also evident in the question they were asked about the importance of systematic reviews in clinical decision-making, with 90% of them thinking it was essential in EBM before taking the course and a full 100% after the course.

### Beliefs about EBM reality in Syria

The questionnaire included questions about the problems facing proper EBM practice in Syria. The fact that the undergraduate medical curriculum at Damascus University lacks a module for teaching EBM was clearly reflected in the students’ answers. Forty-seven (94%) of the students stated that this was one of the main obstacles in bringing EBM practice in Syria up to international standards. When asked about the importance of having role models in EBM practice among faculty teaching staff, 46 (92%) of the students said this was important or very important. The students were asked about importance of EBM infrastructures. Thirty-five (70%) of them thought that the presence of a librarian was important or very important; 47 (94%) thought that subscription to medical journals was important or very important; and 29 (58%) thought that the current information technology hardware were insufficient.

At the end of the course, 74% of the students rated our course as good or excellent, and 42% reported that more material should be included in future courses.

## Discussion

This study was designed to measure how effective it would be to expose undergraduate medical students to EBM principles and practice, in terms of developing a positive attitude toward EBM and improving EBM knowledge and skills. There are different modules of teaching EBM to students as reported in EBM literature; these modules range from workshops, morning reports and journal clubs, to the systems-based method, the problem-based method, or the integration in the curriculum in the basic science and clinical years [22-25]. In our study, lectures and workshops we used as a module of EBM teaching, which has been shown to be an effective educational method in terms of improving students’ skills [26,27].

The pre- and post-course results of our study indicate that our EBM workshops positively changed undergraduate medical students’ beliefs and attitudes toward EBM and improved their perceived knowledge and skills about EBM. Additionally, students have shown a great interest and desire to incorporate teaching of EBM in their undergraduate curricula.

Teaching EBM in Syria is hindered by several challenges. One of them is the need of ‘role-models’ for the students to see EBM being applied on the wards by a variety of senior clinicians and the need to promote EBM ‘culture’ in the clinical setting. Changing the behavior of such clinicians by urging them to use EBM methods more frequently on rounds will have a significant impact on their junior doctors as well as the students, which in turn will have a direct effect on improving the quality of care in the future. This behavior change is not too lofty of a goal, especially when keeping in mind that the ultimate goal of those clinicians and educators is to enable learners to use EBM in their daily practice of medicine and view literature searches as a fundamental skill, similar to history-taking and physical examination [28]. It is sometimes difficult to convey the importance and impact of EBM to preclinical students, hence the need and importance of conducting foundation courses, like ours, that can familiarize students with the key concepts and motivate them to learn and practice EBM.

The teaching and practice of EBM obviously needs certain resources. Rapid and convenient access to valid and relevant evidence on a portable computing device has been shown to improve learning in evidence-based medicine, increase current and future use of evidence, and boost students' confidence in clinical decision making [29]. However, the Faculty of Medicine at Damascus University lacks the technical infrastructure for providing proper means to provide such mechanism of up-to-date practice for undergraduate and postgraduate medical students. The main pillars for such infrastructure are unavailable, namely: a librarian, subscription to online or hardcopy international medical journals, and too few computers relative to the number of students on campus or in affiliated hospitals. In fact, up to the date of the study, there was no access to online medical literature at the Faculty of Medicine or any of its six affiliated hospitals. Therefore, students and residents at these hospitals could only access abstracts or free full articles, and PubMed was the only free biomedical database available.

The main limitation of our study is the students’ self-assessment of their attitudes, rather than direct measurement of behavior, and thus it is not known whether those self-reported changes translated into actual changes in behavior. This study also lacks data on whether improvements in attitudes were sustained over time. Other limitations included small sample size and inability to provide internet access for all students to search for evidence. Alternatively, to teach the students online search, we asked them to build a search strategy, and we applied the search strategy on a big screen with internet access.

In Syria, EBM skills have traditionally not been covered in undergraduate or postgraduate education. Based on our study, integrating EBM teaching in undergraduate medical curriculum in Syria would be feasible and might imply a better EBM practice in the future. Assigning a clinical physician to every group of students as an EBM advisor, whose role is to encourage the teaching and learning of EBM, would greatly facilitate an inclusion of EBM in undergraduate medical curricula. Medical Schools and their decision-makers have the complete responsibility of introducing EBM principles to medical students in order for them to provide evidence-based health care in light of patients’ values and preferences. After all, we believe that there are a significant number of Syrian medical professionals with a solid EBM background who might be good candidates of future EBM tutors. A properly prepared and delivered teaching module of EBM would constitute a cornerstone in lifting EBM reality in Syria up to needed standards and would significantly raise medical students’ awareness and skills to enable them to become efficient physicians that rely on evidence in their health practice [25].

## Conclusions

To date, EBM skills have not been traditionally covered in undergraduate or postgraduate education in Syria. The road to a better EBM reality in Syria starts with teaching EBM in medical school and developing the proper environment to facilitate transforming current medical education and practice to an evidence-based standard in Syria. Based on our study, integrating EBM teaching in undergraduate medical curriculum in Syria would be feasible and might imply a better EBM practice in the future. This integration would constitute the cornerstone in lifting EBM in Syria up to the needed standards and would enable medical students to become efficient physicians that rely on evidence in their health practice.

## Competing interests

The authors declare that they have no competing interests.

## Authors’ contributions

FA designed the study, contributed to data collection and analysis, contributed to course mentoring, and drafted the manuscript. BF contributed to data analysis and course mentoring, and helped draft the manuscript. RH helped draft the manuscript and contributed to course mentoring. MBS contributed to data collection and course mentoring, and helped draft the manuscript. M Fares contributed to data collection and course mentoring, and helped draft the manuscript. IA contributed to design of the study and course mentoring. AS contributed to data collection and course mentoring, and helped draft the manuscript. M Ferwana conceived the idea of the study and helped draft the manuscript. All authors read and approved the final manuscript.

## Disclaimer

No research funding was received by any of the authors. And to the author’s knowledge, no conflict of interest, financial or other, exists. All those listed as authors are qualified for authorship; and all who are qualified for authorship are listed as authors in the byline.
